# Fast Computing Betweenness Centrality with Virtual Nodes on Large Sparse Networks

**DOI:** 10.1371/journal.pone.0022557

**Published:** 2011-07-27

**Authors:** Jing Yang, Yingwu Chen

**Affiliations:** Department of Information Systems and Management, National University of Defense Technology, Changsha, China; Université de Lausanne, Switzerland

## Abstract

Betweenness centrality is an essential index for analysis of complex networks. However, the calculation of betweenness centrality is quite time-consuming and the fastest known algorithm uses 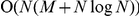 time and 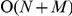 space for weighted networks, where 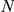 and 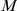 are the number of nodes and edges in the network, respectively. By inserting virtual nodes into the weighted edges and transforming the shortest path problem into a breadth-first search (BFS) problem, we propose an algorithm that can compute the betweenness centrality in 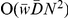 time for integer-weighted networks, where 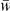 is the average weight of edges and 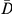 is the average degree in the network. Considerable time can be saved with the proposed algorithm when 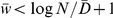, indicating that it is suitable for lightly weighted large sparse networks. A similar concept of virtual node transformation can be used to calculate other shortest path based indices such as closeness centrality, graph centrality, stress centrality, and so on. Numerical simulations on various randomly generated networks reveal that it is feasible to use the proposed algorithm in large network analysis.

## Introduction

Networks, especially complex networks, have been extensively studied during the last decade [1]–[3]. Owing to the ability to gather and analyze large scale data using computers and communication networks, it is quite common to see studies on networks with millions of vertices (nodes) nowadays. The shift of studies from simple small graphs to large complex networks have increasingly contributed new findings of critical phenomena and development of theories in many fields, such as the scale-free distribution of network degrees [4], [5], burstness of human behaviors [6], vulnerability of internet networks [7], [8], and so on [1]–[3], [9].

However, the computation of several network properties, such as the shortest paths, betweenness centrality and closeness centrality, are hindered by the large computation complexity [3], [10]. As a result, many large-scale networks are regarded as unweighted when the above measures are reported [2], [3]. Large efforts have been made to improve the efficiency of algorithms for calculating those network properties [10], [11]. Take the betweenness centrality, for example [12], [13]: for a weighted network 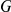 with 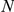 nodes and 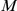 edges, the naive algorithm requires 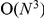 time and 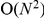 storage, regardless of the algorithms implemented to find the shortest paths. A much faster algorithm proposed by Brandes [14], on the other hand, can calculate the betweenness centrality in 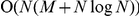 time and 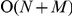 space when the shortest paths are calculated by Dijkstra's algorithm implemented with a Fibonacci heap. Parallel algorithms are also proposed to improve the efficiency for the calculation of betweenness centrality [10], [11], [15]–[21]: for example, Bader and Madduri [10] proposed a betweenness centrality algorithm on a high-end shared memory symmetric multiprocessor and multithreaded architectures, with which is “possible” to achieve the computation in 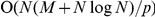 time with access conflicts, where 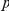 is the number of processors used. However, the parallel algorithms requires much more complex programming and are highly dependent on the hardwares: for example, in Bader and Madduri's study [10], they used an IBM p5 570 on 16 processors and utilized 20GB RAM. These equipments are obviously not adaptable for general network researchers.

To circumvent the difficulties in calculating betweenness centrality with large time complexity, we propose a new algorithm for integer-weighted networks in this paper. By replacing the weighted edges with connected virtual nodes, the new algorithm computes the betweenness centrality in 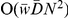 time and 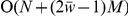 space, with 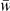 and 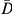 being the average edge weight and average degree of the network, respectively.

## Methods

### The Brandes' Algorithm

Given a network 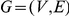, with 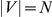 the number of nodes and 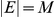 the number of edges, for the purpose of this study, we consider strongly connected networks [22] with no self loops (acyclic). Let 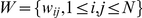 be the weight matrix of 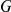, where 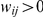 is the weight on edge 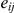. In real practice, 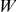 can be distances between airports, information flows between computers, traffic loads between cities, etc.

Let 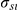 denote the number of shortest paths from node 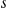 to 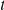, and 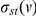 be the number of shortest paths from 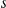 to 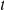 that pass through 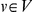, then the betweenness centrality of node 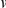 is defined as [13], [14]:
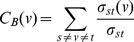
(1)From the definition we can see that betweenness centrality is the sum of the fraction of shortest paths over all pairs of nodes passing through the node, high betweenness centrality indicates that a node can reach others (or be reached by others) with relatively short paths, or the node lies on considerable fraction of shortest paths connecting others. In many fields, the betweenness centrality can be regarded as a measure of the extent to which the node has control over information flowing between others, and it is thus a core index for evaluating the importance of nodes in the network [13], [23]. For example, in the study of networks vulnerability to attacks, the removal of nodes with the highest betweenness centrality is shown to be one of the most harmful strategies that can break down the networks [8].

A straightforward way of calculating the betweenness centrality then use the following steps:

**Step 1** Compute the length and number of shortest paths between all pairs of nodes;

**Step 2** For each node 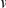, calculate 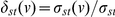 (*pair dependency*) for each pair and sum them up.

Obviously, the complexity of the naive algorithm is dominated by the second step which requires 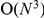 time summation and 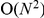 storage of *pair dependencies*. To introduce Brandes' algorithm, we first define the set of predecessors of node 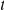 on the shortest paths from 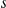:
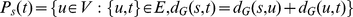
(2)where 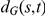 is the distance of the shortest path from 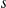 to 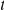. Then the number of shortest paths from 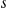 to 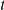 can be calculated as:
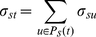
(3)To eliminate the need for explicit summation of all *pair dependencies*, Brandes [14] defines the *dependency* of node 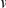 as:
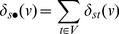
(4)

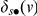
 has the recursive property that
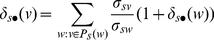
(5)Note that 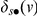 is merely a partial sum of Eq. (1), then the betweenness centrality can be expressed by:
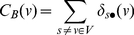
(6)The summation of *pair dependencies* is then reduced to accumulation of *dependencies* defined by Eq. (5). Specifically, given the shortest paths from 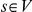 in 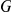, the array storing 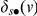 for all nodes can be recursively calculated according to Eq. (5), by traversing the nodes in non-increasing order of their distances from 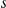. An illustrative algorithm is shown in Algorithm 1. We can see that the calculation for Step 2 is now in 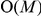 time and 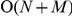 space, then the calculation complexity of betweenness centrality is determined by the shortest path algorithms used in Step 1. Using Dijkstra's algorithm implemented with Fibonacci heap [24], which requires 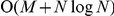 time for the single source shortest path problem [25], the betweenness centrality can be computed by Brandes' algorithm in 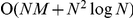 time and 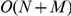 space on weighted networks [14].

### Computing Betweenness Centrality with Virtual Nodes

Brandes' algorithm has greatly reduced the computation burden for betweenness centrality, however, the time complexity is still too high for networks with millions of nodes since the shortest path algorithm would cost a lot of computation time anyway. In this section, we propose a new algorithm that can reduce the time complexity in Step 1, such that the betweenness centrality can be calculated within reasonable time under certain conditions.

#### Replacement of Weighted Edges

Our new algorithm originates from the idea that an integer-weighted network can be broken down into a simple unweighted network with virtual nodes, such that the calculation of shortest paths in Step 1 can be solved as a breadth-first search (BFS) problem.

**Algorithm 1: Brandes' algorithm** [**14**] **.**

1 
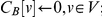


2 for 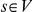 do

3  [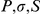] = *single source shortest path algorithm()*

  /*
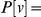
set of predecessors for shortest paths from 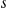 to 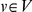;*/

  /*
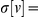
array storing the number of shortest paths from 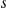 passing through 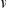; */

  /*
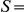
stack storing the distances of nodes from 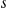 in non-increasing order; */

  /*accumulate dependency from the most distant nodes */

4  
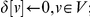


5  while 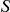 not empty do

6   pop 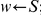


7   for 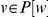 do 
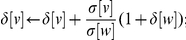


8   if 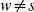 then 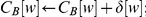


9  end

10 end

**Figure 1:**
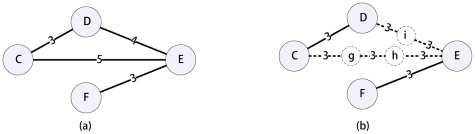
Illustration of representing the weighted network (a) by an unweighted network with virtual nodes (b).

Figure 1 illustrates the representation of an undirected weighted network by an undirected unweighted network with three additional virtual nodes. We can see that edge 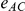 and 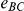 are replaced by 3 and 2 unit edge segments with two and one virtual nodes inserted, respectively. The number of virtual nodes to be inserted on a weighted edge 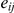, is then 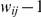.

Let 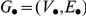 be the unweighted representation of 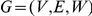 with virtual nodes, where 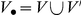 with 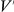 the set of virtual nodes, then the number of virtual nodes in 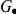, is 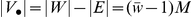, where 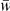 is the average edge weight.

*Virtual Node Based Algorithm for Betweenness Centrality*. Obviously, the insertion of virtual nodes does not change the distances between pairs of nodes in 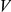 and consequently the number of shortest paths between nodes is the same as in 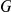. The calculation of shortest paths on 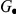 can then be solved by the BFS algorithm, instead of the traditional Dijkstra's algorithm.

However, before applying the BFS on 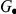 to calculate the betweenness centrality for nodes in 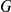, there is at least one problem to be solved: to use the existing theories on summation of *pair dependency* in Algorithm 1, the predecessors of nodes in 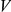 recorded during the shortest path calculation in 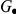, should be kept as the same as if they were calculated by any shortest path algorithm in 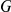. This can be achieved as follows: suppose the BFS finds a shortest path from 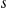 to 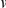: 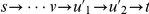, where 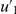, 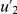 are two virtual nodes inserted on edge 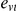, then the predecessor of 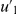, which is 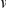, can be passed through 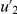 to the next non-virtual node 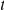:
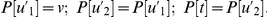
An implementation of the above process is presented in Algorithm 2, the steps for accumulation of *dependency* are identical as the Brandes' algorithm and thereby are omitted.

**Algorithm 2: Virtual node algorithm for betweenness centrality**

1 
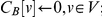


2 for 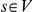 do

3  
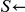
empty stack;

4  
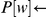
empty list, 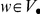;

5  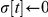, 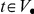; 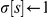;

6  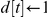, 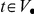; 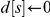;

7  
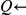
empty queue;

8  enqueue 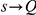;

9  while 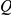 not empty do

10   dequeue 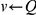;

11   push 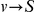;

12   foreach neighbor 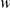 of 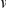 do

13    if 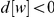 then /*visit 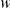 the first time*/

14     enqueue 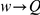;

15     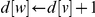;

16    end

17    if 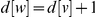 then

18     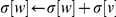;

19     if 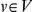 then

20      append 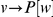;

21      else /*if 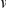 is a virtual node, retrieve the latest non-virtual node as predecessor*/

22      append 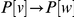;

23     end

24    end

25   end

26  end

27  *accumulate dependency()*/*as shown in Algorithm 1

28 end

Note that in Algorithm 2, we don't need to calculate shortest paths between virtual nodes. The BFS then requires 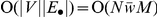 time. For the sake of clarity, let 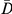 be the average degree of nodes in 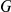 such that 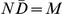, then we have 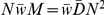. The computation of betweenness centrality with virtual nodes (the VN algorithm), is dominated by the BFS and has a time complexity of 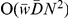, and needs 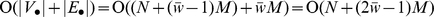 space.

**Figure 2:**
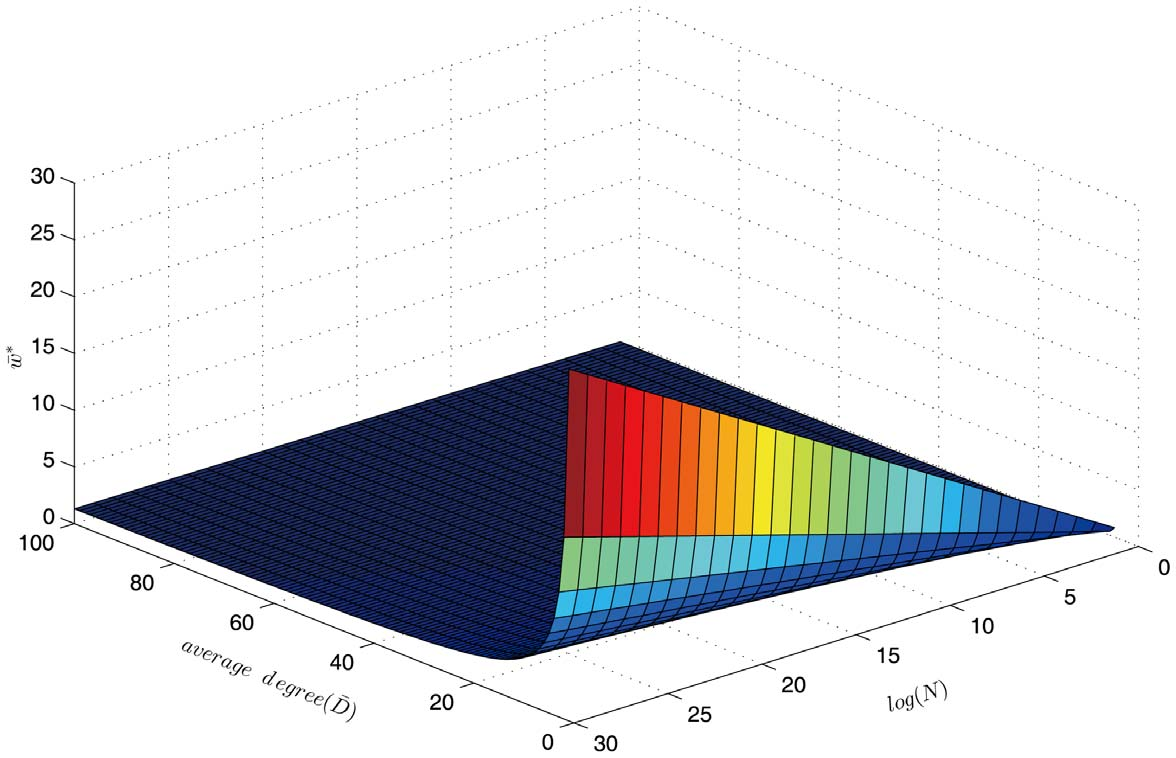
Critical threshold for average weights (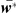) on networks with specified network size (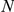) and average degree (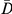).

Compared with Brandes' algorithm, we can see that the VN algorithm will perform better when 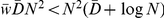, that is, 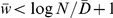. We henceforth denote 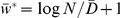 as the critical threshold for the average edge weight on a network; if 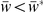, the VN algorithm will be able to calculate the betweenness centrality faster than Brandes' algorithm. Figure 2 shows the distribution of 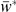 over the domain of combinations of different network sizes and average degrees. We can see that the advantage of the VN algorithm becomes evident when the network is large and sparse, for example, when the network size is 1 million (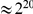), and the average degree is 5, the VN algorithm would be faster for those with 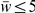; for the same average degree, 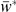 increases to 7 when the network size reaches 1 billion (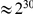). For an average degree of 10, 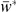 lies beyond 3 for networks larger than 1 million. Note that many large-scale networks are reported to have rather small average degrees; for example, the mobile communication network reported in [26], contains 4.6 million nodes and an average of 3.04 edges. The Internet network [27], math co-authorship network [28], and power grid [29] reported in [1], are found to have average degrees of 3.5–4.1, 3.9 and 2.7, respectively. Networks with low integer weights are also reported in the literature; for example, the neural network of the Caenorhabditis elegans worm [29], the communication network of the online community [30], and the political support network of the US Senate [31], have average edge weights of 3.74, 2.95 and 3.74, respectively.

## Results and Discussion

### Numerical Experiments

To evaluate the algorithms, we generate scale-free networks [32] with different network sizes and edge weights, and the execution time of VN algorithm and Brandes' algorithm are then tested on these networks. Algorithms are coded in C and run on a PC with an Intel Core 2 Quad CPU (2.66 GHz, 6 Mb) and 6 Gb of RAM, all the following reported running times are the average of 100 simulations.

**Figure 3:**
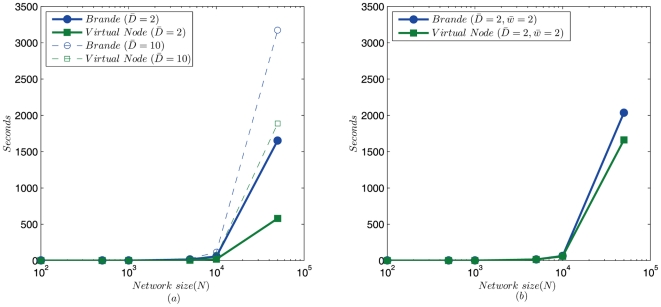
Running time of the VN algorithm and Brandes' algorithm. (a) Networks with average degree 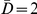 and 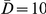, 1% of the network edges are weighted with 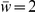; (b) Networks with average degree 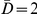, all edges are weighted with 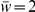.

It is intuitive that when seldom edges in the network are weighted, the VN algorithm will calculate the betweenness centrality approximately as fast as the BFS, which is much faster than the Brandes' algorithm. For example, when the network size is 100,000 and we set the average degree as 2 and take 1000 edges to be weighted with random numbers generated from 1 to 10, the execution time for Brandes' algorithm is 8460 seconds, while the VN algorithm needs only 3830 seconds, which is around 1.3 hours faster than the Brandes' algorithm. Since when 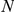 becomes large, we have 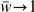, more time can be expected to be saved in larger networks with fixed number of weighted edges. We calculated the VN and Brandes' algorithm on networks with 1% of edges being weighted as 2, and the execution times are presented in Figure 3(a). We can see that the difference in execution time become larger when the network size increases. When the network size is 50,000, the VN algorithm is 3 and 1.5 times faster than the Brandes' algorithm, for average network degrees of 2 and 10, respectively.

The above results reveal that the VN algorithm is much faster on large sparse networks with limited number of weights. However, we should note that the VN algorithm is quite sensitive to the average degree and weight sum of the network, for any network with 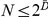, the VN algorithm will not outperform Brandes' algorithm as long as 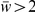. To illustrate the sensitivity of the VN algorithm, we run algorithms on networks with 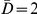 and 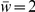, and the difference in running times between the two algorithms decreases quickly as expected (Figure 3(b)).

### Discussion

By replacing the weighted edges with connected virtual nodes, we propose the VN algorithm to calculate the betweenness centrality in weighted networks with the BFS rather than shortest path algorithms. The VN algorithm uses 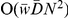 time and 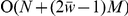 space. Theoretically, the VN algorithm outperforms the Brandes' algorithm when 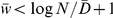, indicating that when the average edge weight is low, considerable time can be saved on large sparse networks. The simulation study confirms that when 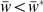, more time can be saved when the network grows large.

We should note that the VN algorithm is quite sensitive to the density and weight of the networks, it can hardly outperform the Brandes' algorithm when the network is dense and weighted with large values. What's more, the theoretical threshold value 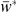, could be even lower in practice since the VN algorithm requires more space. Despite these limitations, given the evidences that large-scale networks in real life are mostly sparse, and the BFS is much easier to implement than the Fibonacci heap based shortest path algorithms, the VN algorithm is expected to be able to save analysis time in many scenarios. Moreover, the VN algorithm can easily be generalized to calculate other shortest path based network properties, such as closeness centrality [33], graph centrality [34], stress centrality [35], and so on. We henceforth recommend that network researchers to use the VN algorithm when the studied network is large, sparse, and lightly weighted, but continue to use the Brandes' algorithm otherwise.

### Supporting Information

Both the Brandes' algorithm and the VN algorithm are written in C and are available upon request from the author.
